# Do racial and ethnic disparities in following stay-at-home orders influence COVID-19 health outcomes? A mediation analysis approach

**DOI:** 10.1371/journal.pone.0259803

**Published:** 2021-11-11

**Authors:** Songhua Hu, Weiyu Luo, Aref Darzi, Yixuan Pan, Guangchen Zhao, Yuxuan Liu, Chenfeng Xiong

**Affiliations:** 1 Maryland Transportation Institute (MTI), Department of Civil and Environmental Engineering, University of Maryland, College Park, MD, United States of America; 2 Shock Trauma and Anesthesiology Research (STAR) Center, School of Medicine, University of Maryland, Baltimore, MD, United States of America; Tongji University, CHINA

## Abstract

Racial/ethnic disparities are among the top-selective underlying determinants associated with the disproportional impact of the COVID-19 pandemic on human mobility and health outcomes. This study jointly examined county-level racial/ethnic differences in compliance with stay-at-home orders and COVID-19 health outcomes during 2020, leveraging two-year geo-tracking data of mobile devices across ~4.4 million point-of-interests (POIs) in the contiguous United States. Through a set of structural equation modeling, this study quantified how racial/ethnic differences in following stay-at-home orders could mediate COVID-19 health outcomes, controlling for state effects, socioeconomics, demographics, occupation, and partisanship. Results showed that counties with higher Asian populations decreased most in their travel, both in terms of reducing their overall POIs’ visiting and increasing their staying home percentage. Moreover, counties with higher White populations experienced the lowest infection rate, while counties with higher African American populations presented the highest case-fatality ratio. Additionally, control variables, particularly partisanship, median household income, percentage of elders, and urbanization, significantly accounted for the county differences in human mobility and COVID-19 health outcomes. Mediation analyses further revealed that human mobility only statistically influenced infection rate but not case-fatality ratio, and such mediation effects varied substantially among racial/ethnic compositions. Last, robustness check of racial gradient at census block group level documented consistent associations but greater magnitude. Taken together, these findings suggest that US residents’ responses to COVID-19 are subject to an entrenched and consequential racial/ethnic divide.

## 1. Introduction

Starting from December 2019, the Coronavirus disease 2019 (COVID-19) pandemic has infected millions of people and deteriorated into one of the worst worldwide public health crises seen in decades [1]. The United States has stubbornly remained the epicenter of the COVID-19 pandemic since the spring of 2020, reporting the highest number of total infections and deaths worldwide. Non-pharmaceutical interventions such as travel bans and stay-at-home orders have been enacted in the US, national and local. However, governments lifted those policies progressively since May 2020 [2–4]. The COVID-19 infections and deaths also experienced subsequent waves of rising and fall as the pandemic progressed. Quantifying the impact of non-pharmaceutical measures on reducing human mobility and COVID-19 infections and deaths, and identifying determinants associated with human mobility changes and COVID-19 health outcomes are crucial for assessing the implemented interventions, tailoring policies to the needs of targeted socioeconomic groups, and eventually curb the spread of COVID-19 and prevent future outbreaks.

Before the successful deployment of vaccinations and medications, stay-at-home order is one of the most effective means to contain the dissemination of COVID-19. Numerous studies have shown that stay-at-home orders can help limit the spread of the virus [5–7]. However, concerns arise regarding that compliance with stay-at-home orders might be a manifestation of socioeconomic privilege [2, 8–13]. Aided by the real-time location-based service data collected from a range of regions, researchers were witnessing a stark socioeconomic disparity in following stay-at-home orders worldwide [8–10, 12, 14–17]. Regions with higher median income, fewer “essential” workers, fewer racial/ethnic minorities, and higher autonomy to work from home were reducing mobility significantly more [8–10, 18–20]. In the US, partisanship is another main factor related to compliance with stay-at-home orders. Several studies claimed that Republican-leaning counties exhibited considerably lower compliance with stay-at-home orders than Democrat-leaning counties [2, 21, 22]. On the other hand, socioeconomic gradient also exists in COVID-19 health outcomes. Mounting evidence suggested disadvantaged and vulnerable social groups, especially the racial/ethnic minorities, the elders, and the low-income “essential” workers, were bearing the brunt of the infection and death toll [23–25].

Racial/ethnic (abbreviated as racial) disparities are among the top-selective underlying determinants associated with the disproportional impact of COVID-19 pandemic on human mobility and COVID-19 outcomes. Underrepresented minorities, particularly African Americans [24–30] and Hispanics/Latinos [23, 28, 30, 31], were at higher vulnerability to the COVID-19 infection and death. Latest studies demonstrated that even after adjusting for numerous confounders such as socioeconomics, demographics, occupation, healthcare resource, and pre-existing health issues, regions with more racial minorities still exhibited higher infection and/or death rates [28, 31]. On the other hand, regarding staying home behaviors, it is currently inconclusive on which racial group engaged more: Some researchers documented African American-leaning regions reduced more in human mobility [18, 32, 33] while others stated White-leaning regions reduced more [2, 8, 24, 34, 35]. When it comes to the intertwined relationships among racial make-up, socioeconomic status, healthcare resources, and partisanship, such controversy highlights the need to disentangle the racial disparities via a well-designed structure.

We saw previous COVID-19 related studies examining the socioeconomic disparities and their impacts. However, current studies have solely focused on the racial disparities are limited. Also, few studies have jointly analyzed the racial differences in human mobility and COVID-19 health outcomes under one model framework. It remains unclear whether and to what extent racial differences in human mobility could translate into COVID-19 side-effects. Does human mobility mediate the infection and fatality rates of different racial groups to the same level? Do racial groups more assiduously adhering to stay-at-home orders also exhibit lower infection and case-fatality rates? Moreover, do those racial differences hold robust under different mobility and health metrics or after adjusting for multiple confounders? All of these questions remain unclear and deserve further exploration.

This study examined two issues: the racial differences in human mobility and COVID-19 health outcomes via mediation analysis. In particular, we assess staying home behaviors via two metrics: percentage of visit change using the volume in the same week of the year in 2019 as baseline (abbreviated as visit change (%)), and percentage of people staying home (abbreviated as staying home (%)). We use the number of COVID-19 cases per 100,000 population (abbreviated as cases/100,000) and the number of COVID-19 deaths per 100 confirmed cases (aka case-fatality ratio, abbreviated as deaths/100 cases) to calibrate the COVID-19 health outcomes. Methodologically, this study followed three steps: First, confirmed the existence of racial disparities by using t-tests to show the differences of human mobility and COVID-19 health outcomes among racial quintiles. Second, controlled for socioeconomics, demographics, occupation, partisanship, and state effects to see whether racial disparities attenuated or disappeared. Last, linked the racial make-up with COVID-19 health outcomes via their human mobility changes to explore whether their differences in human mobility could mediate COVID-19 health outcomes accordingly.

Our analysis contributes a complementary contextual and statistical component building on previous work. Firstly, it analyzes racial disparities topics during the pandemic leveraging two-year large-scale spatiotemporal human mobility and health data across the contiguous US. Secondly, it jointly investigates the racial differences in human mobility and COVID-19 health outcomes via structural equation modeling. Thirdly, it highlights the intertwined risk factors of racial make-up, socioeconomics, demographics, occupation, partisanship, and state effects and quantifying their associations with four mobility and health metrics. Last but not the least, it tests the robustness of results at both county level and census block group level. Our findings are reliable sources to tailor health-related policies. They are helpful when considering the needs of specific socioeconomic groups, to reduce the stay-at-home privilege gap, and to help halt the transmission of the virus.

## 2. Research design

### 2.1. Preliminary analysis

We conducted a set of preliminary analyses before the model building. First, spatiotemporal distributions of county-level human mobility and COVID-19 health outcomes stratified by racial quintile were depicted. An unpaired t-test was employed to compare the mean value of the four metrics between racial quintiles. For each racial quintile, we estimated the mean value of the four metrics. And we compared it with the mean value in the corresponding lowest quintile. The preliminary analysis did not control for any confounders. Therefore, it would be inappropriate to interpret the observed racial gradient as causal in this step. We developed a series of supplemental analyses at the census block group (CBG) level (S1 Table). In addition, we performed the pairwise analysis with all variables to understand the bivariate relationships between exogenous variables and four metrics. The same applies to the intercorrelations among exogenous variables. Last, variable and observation selection were conducted based on variance inflation factor (VIF), Cook’s distance, and stepwise regression to determine the optimal data set.

### 2.2. Mediation analysis

**Fig 1:**
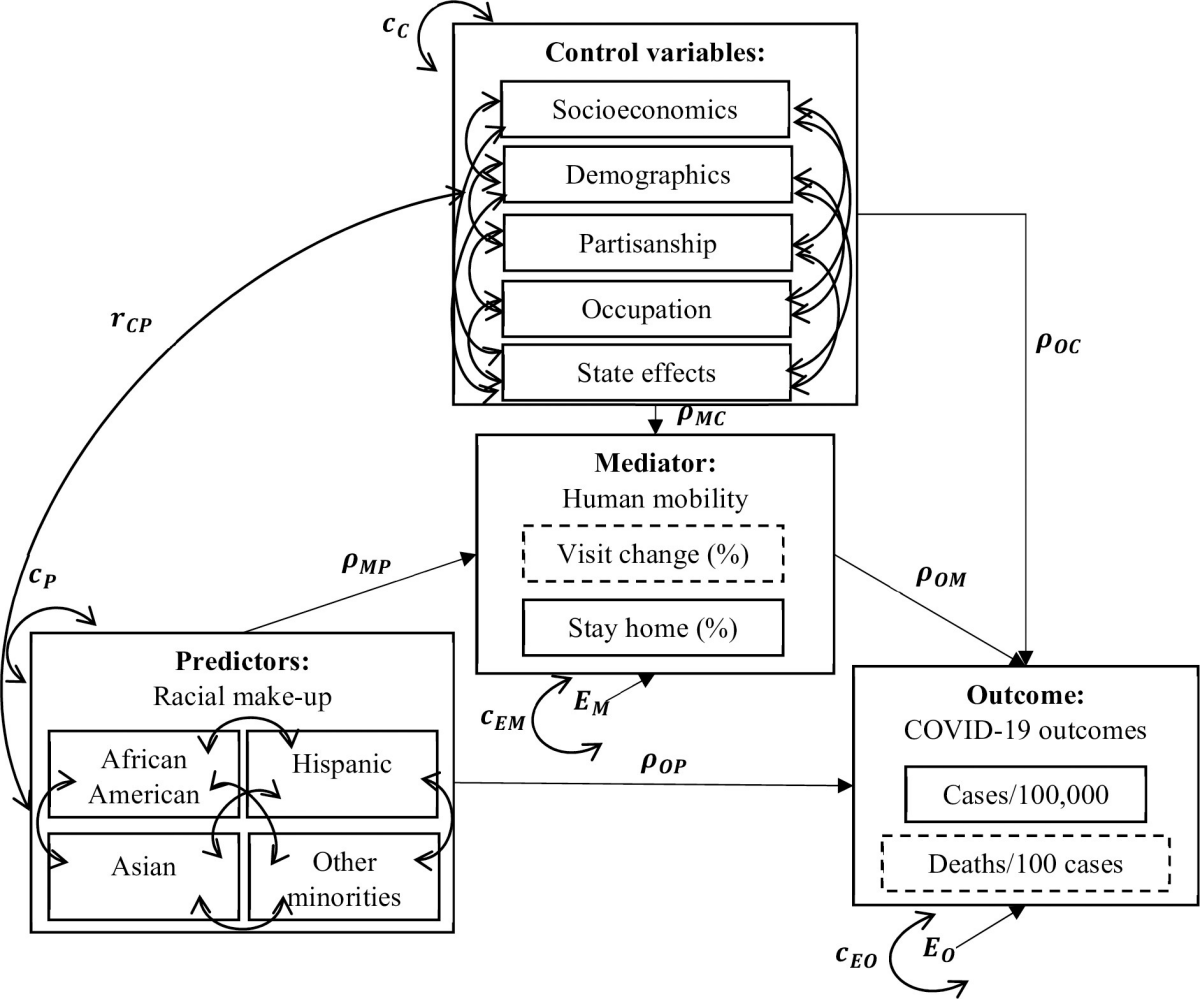
Conceptual diagrams for mediation analysis. *c*_*EM*_ means the variance of the error term of mediators; ***c***_***EO***_ means the variance of the error term of outcomes; ***c***_***C***_ means the variance of controls; ***c***_***P***_ means the variance of predictors; ***r***_***CP***_ refers to the covariance matrix between control variables and predictors.

Mediation analysis is helpful to understand the underlying mechanism or process by which some hypothetical causal variable influences an outcome through at least one mediator [36]. In our case, we aim to understand whether racial differences in human mobility influence their COVID-19 health outcomes. Fig 1 depicts the conceptual diagram for our mediation analysis, where predictors refer to four racial compositions, mediator refers to one of the human mobility metrics, outcome refers to one of the COVID-19 health outcomes, and control variables include other confounders.

We assumed: 1) Exogenous variables, including controls and predictors, covary with each other; 2) Predictors affect outcome both directly and indirectly through mediators; 3) Control variables exert direct effects on endogenous variables, including mediators and outcomes; 4) There exists unobserved factors affecting all endogenous variables; 5) When estimating unstandardized path coefficients, the variances of all locally exogenous variables were considered; 6) State fixed effects were controlled by state dummy variables in one-hot encoding; 7) To avoid some variables generating extremely larger observed variances than others, we scaled those variables with greater magnitude by dividing a constant before model fitting and resized their unstandardized estimations back by multiplying that constant. We built four structural equation models (SEMs) with different human mobility and COVID-19 outcome metrics. The mediators and outcomes can be calculated by Eqs (1 and 2), and structural equations are listed as Eqs (3 and 4):

$$V_{i}\%=\frac{\sum_{t=9}^{T}v_{i,\;t}-\sum_{t=9}^{T}{\ddot{v}}_{i,t}}{\sum_{t=9}^{T}{\ddot{v}}_{i,t}},S_{i}\%=\frac{\sum_{t=9}^{T}s_{i,t}}{\sum_{t=9}^{T}d_{i,t}}$$ (1)

$$C_{i}=100000\frac{\sum_{t=9}^{T}c_{i,t}}{P_{i}},\;F_{i}\%=\frac{\sum_{t=9}^{T}f_{i,t}}{\sum_{t=9}^{T}c_{i,t}}$$ (2)

$$M=\rho_{MP}P+\rho_{MC}C+E_{M},\;M\in V_{i},S_{i}$$ (3)

$$O=\rho_{OP}P+\rho_{OM}M+\rho_{OC}C+E_{O},\;O\in C_{i},F_{i}$$ (4)

where:

1. *V*_*i*_, *S*_*i*_ are the average percentage of visit change and staying home from 1 March (week of year = 9) to 31 December 2020 (week of year *T* = 52) in county *i*; *C*_*i*_, *F*_*i*_, are accumulative number of COVID-19 cases/100,000 and deaths/100 cases from 1 March to 31 December 2020 in county *i; v*_*i*,*t*_ is the POI visit counts in county *i* in week *t* of 2020; ${\ddot{v}}_{i,t}$ is the POI visit counts in county *i* in week *t* of 2019; *s*_*i*,*t*_ is the number of people staying home in county *i* in week *t*; *d*_*i*,*t*_ is the number of observed mobile devices in county *i* in week *t*; *c*_*i*,*t*_ is the number of confirmed new cases in county *i* in week *t*; *f*_*i*,*t*_ is the number of fatalities in county *i* in week *t*; *P*_*i*_ is the total population of county *i*;
2. *M* is a mediator from *V*_*i*_, *S*_*i*_; ***P*** is the set of racial compositions; ***C*** is the set of control variables; *O* is an outcome from *C*_*i*_, *F*_*i*_; ***ρ***_***MP***_, ***ρ***_***OP***_, *ρ*_*OM*_ are the path coefficients between predictor and mediator, between predictor and outcome (aka direct effect), and between mediator and outcome, respectively; ***ρ***_***MC***_ and ***ρ***_***OC***_ are path coefficients of control variables; *E*_*M*_ and *E*_*O*_ are the error terms. Indirect effects were deduced from ***ρ***_***MP***_*ρ*_*OM*_. 95% CIs of direct and indirect effects were obtained through 1000 bootstraps.

### 2.3. Data and variable description

#### Predictors

Racial make-up information came from the most recent 5-year (2015–2019) American Community Survey (ACS) of the US Census Bureau. The racial compositions (e.g. White, Black or African American, Asian, American Indian or Alaska Native, Native Hawaiian or Pacific Islander, and others) and ethnicity (e.g. Non-Hispanic or Latino and Hispanic or Latino) can be complicated over 12 racial/ethnical classifications. However, we mainly chose the top four highest regarding their percentage of the total population, which is also widely used by the US Census Bureau. One caveat is that Hispanics may be of any race as Whites, African Americans, Asians, and other minorities. Considering a majority (65.5%) of Hispanics identified themselves as Whites, we involved Non-Hispanic White (abbreviated as White) in our study as a reference to help distinguish the differences between Hispanics and Whites.

#### Mediators

We assessed residents’ stay-at-home behaviors using mobile devices data from SafeGraph [37], a company that aggregates anonymized geo-tracking data from ~4.4 million point-of-interest (POIs) in the US. SafeGraph data is one of the largest data sources in North America for tracking human movement during the COVID-19 pandemic [8, 10]. Various previous studies have also shown that the SafeGraph is highly dovetailed with multiple mobility data sources like Google [8] and PlaceIQ [9]. In addition, SafeGraph has completed validations that suggested their data are consistent with Census data [38]. For example, it does not systematically over or underrepresents individuals among different racial make-up or socioeconomic statuses.

We used the Weekly Places Patterns (v2) datasets [37] to calculate the county-level & CBG-level percentage change in POI visits. The Social Distancing Metrics v2.1 [37] is used to estimate the county-level & CBG-level percentage of people staying home, which are the devices staying throughout the day within ~150 meters of their common nighttime locations. All location data were de-identified. And they contain no private personal information. To prevent double-counting, we removed all parent POIs [8]. To address those low-sample biases, we cleared some of the counties manually because their average number of observed devices is smaller than 5% of the county population.

#### Outcomes

The cases/100,000 and deaths/100 cases were from COVID-19 Data Repository by the Center for Systems Science and Engineering (CSSE) at Johns Hopkins University [1]. Their dataset includes daily cumulative counts of county-level COVID-19 infections and fatalities across the US.

#### Control variables

Control variables included socioeconomics, demographics, occupation, partisanship, and state fixed effects. Data sources of control variables are as follows: Socioeconomics, demographics, occupation, and state fixed effects were from the 2015–2019 American Community Survey (ACS) 5-year estimates, partisanship was from the 2016 presidential election result from the MIT election lab [39]. After variable selection, control variables include: 1) Socioeconomics: median household income, GINI coefficient, and % of residents with no health insurance coverage; 2) Demographics: % of male, % of residents aged 65 years and over, population density, and rurality (central (reference), outlying, and rural); 3) Occupation: % of manufacturing, % of healthcare and social assistance, % of administration, business support and waste management services, % of retail and wholesale, % of transportation, warehousing, and utilities, % of educational services, and % of accommodation, food, arts, entertainment, and recreation services. 4) Partisanship: % of Democrats in 2016 presidential candidate vote totals, with the % of Republicans setting as reference.

### 2.4. Variable selection and pairwise analysis

Variable selection was performed to determine the optimal variable set. The variance inflation factor (VIF) was first calculated to test the multicollinearity, and VIFs greater than 5 were excluded. Then, a forward stepwise regression was used to select the optimal independent variables based on the smallest AIC. We summarized variables in the final models in Table 1. To eliminate the effects of outliers, we excluded influential outliers by removing the observations if their Cook’s distance exceeded the cutoff value 4/(*n*−*k*−1), where *n* is the sample size, and *k* is the number of locally exogenous variables in each structural equation [40].

**Table 1:** Summary of county-level variables.

		Description	Mean	St.d.	Median	Min.	Max.
**Mediators**							
Visit change (%)		Average percentage change in POI visits during 1 March to 31 December 2020 using the volume in the same week of year in 2019 as baseline, in %	-12.13	13.55	-10.96	-63.03	35.62
Staying home (%)		Average percentage of people staying home during 1 March to 31 December 2020, in %	27.67	4.21	27.40	13.90	42.64
**Outcomes**							
Cases/100,000		Cumulative number of COVID-19 cases per 100,000 population by the end of 2020	6454.10	2688.11	6236.96	0.00	21302.00
Deaths/100 cases		Cumulative number of COVID-19 deaths per 100 confirmed cases by the end of 2020	1.79	1.09	1.57	0.00	8.43
**Predictors**							
Racial/ ethnic groups	*White*	*Percentage of Non-Hispanic White populations*, *in % (Reference)*	76.77	19.65	84.01	0.69	99.59
	African American	Percentage of African American populations, in %	9.16	14.57	2.34	0.00	87.23
	Asian	Percentage of Asian populations, in %	1.28	2.26	0.62	0.00	36.47
	Hispanic	Percentage of Hispanic/Latino populations, in %	9.39	13.83	4.22	0.00	99.17
	Other minorities	Percentage of American Indian and Alaska Native alone, Native Hawaiian or other Pacific Islander, two or more races, and other populations, in %	6.12	7.75	3.91	0.00	89.76
**Control variables**							
Occupation	Administration	Percentage of administration, business support, and waste management services, in %	3.27	1.40	3.23	0.00	15.69
	Manufacture	Percentage of manufacturing industry, in %	12.40	7.07	11.45	0.00	46.39
	Retail	Percentage of retail trade and wholesale trade, in %	13.63	2.60	13.72	1.27	30.63
	Transportation	Percentage of transportation, warehousing, and utilities, in %	5.59	1.99	5.35	0.00	21.85
	Education	Percentage of educational services, in %	9.33	3.20	8.73	0.81	36.12
	Health Care	Percentage of healthcare and social assistance, in %	13.89	3.35	13.85	0.75	38.15
	Accommodation & Food	Percentage of accommodation, food, arts, entertainment, and recreation, in %	8.30	3.54	7.93	0.00	40.49
Socioeconomic	GINI	A measure of statistical dispersion to represent the income inequality, from 0 (maximal inequality) to 1 (perfect equality)	0.45	0.04	0.44	0.32	0.71
	Median Income	The median household income (in 2019 Inflation-Adjusted Dollars), in $10^4^/household	5.33	1.40	5.17	2.15	14.23
	Without Insurance	Percentage of residents with no health insurance coverage, in %	9.53	4.91	8.65	0.67	40.91
Demographic	Population Density	Population density, in 10^4^ persons/sq. mile	0.02	0.07	0.00	0.00	1.45
	Rurality	1: Central (41.22%, *Reference*); 2: Outlying (17.78%); 3: Rural (41.00%)	-	-	-	-	-
	Age over 65	Percentage of residents 65 years and over, in %	18.87	4.58	18.48	6.62	56.71
	Male	Percentage of male, in %	50.03	2.26	49.63	42.81	72.72
Partisanship	Democrat	Percentage of Democrats in 2016 presidential candidate vote totals, in %	31.35	14.96	28.24	3.14	88.13
	*Republican*	*Percentage of Republicans in 2016 presidential candidate vote totals*, *in % (Reference)*	63.44	15.42	66.38	8.40	96.03

**Note:** Variables in *Italic* were excluded from the models as reference variables.

An important caveat here is that most variables were calculated at an aggregate (county) level instead of an individual (residents) level. Thus, conclusions drawn from this study should not be extrapolated to individuals due to the potential ecological fallacy. Another concern is considering the modifiable area unit problem (MAUP), which postulates that different aggregation units may lead to different modeling results [41]. This concern cannot be ignored, especially in the US with significant residential segregation. A county-level analysis may gloss over socioeconomic disparities in human mobility and COVID-19 health outcomes existing at a more localized level [42]. However, due to the inaccessibility of finer-grained data in both outcomes (i.e. cases/100,000 and deaths/100 cases) and some control variables (e.g. GINI, partisanship, rurality), we employed the county as our spatial unit, which was the finest data that can be acquired for all variables. For robustness check, we also partially conducted our research at the CBG level with those variables that can be obtained at the CBG level.

The pairwise Pearson correlation heatmap reordered by the dendrogram was depicted in S1 Fig. We found *Democrat* presented the strongest positive correlation with human mobility (i.e. positively related to *Staying home (%)* and negatively related to *Visit change (%)*), followed by *Asian* and *Median Income*. Conversely, *Republicans* presented the strongest negative correlation with human mobility, followed by *Rurality*. Compared with human mobility metrics, COVID-19 health outcomes showed lower correlations with other variables, with the most significant absolute Pearson coefficient lower than 0.4. Specifically, cases/100,000 exhibited the strongest negative correlation with *Staying home (%)*, followed by *Administration*, and presented the most positive relationship with *Visit change (%)*. Deaths/100 cases were negatively associated with *White* and *Median Income* and positively associated with *African American* and *Age over 65*. We also noticed the dendrogram highlighted four main clusters regarding their interrelationships: 1) *GINI*, *African American*, and *Deaths/100 cases*; 2) *Other Minorities*, *Hispanic*, and *Without Insurance*; 3) *Population Density*, *Administration*, *Food*, *Democrat*, *Asian*, *Staying home (%)*, and *Median Income*; 4) *Rurality*, *Age over 65*, *Visit Change (%)*, *Republican*, and *White*. Variables within each cluster were strongly and positively interplayed with each other.

## 3. Results

### 3.1. Spatiotemporal distributions

**Fig 2:**
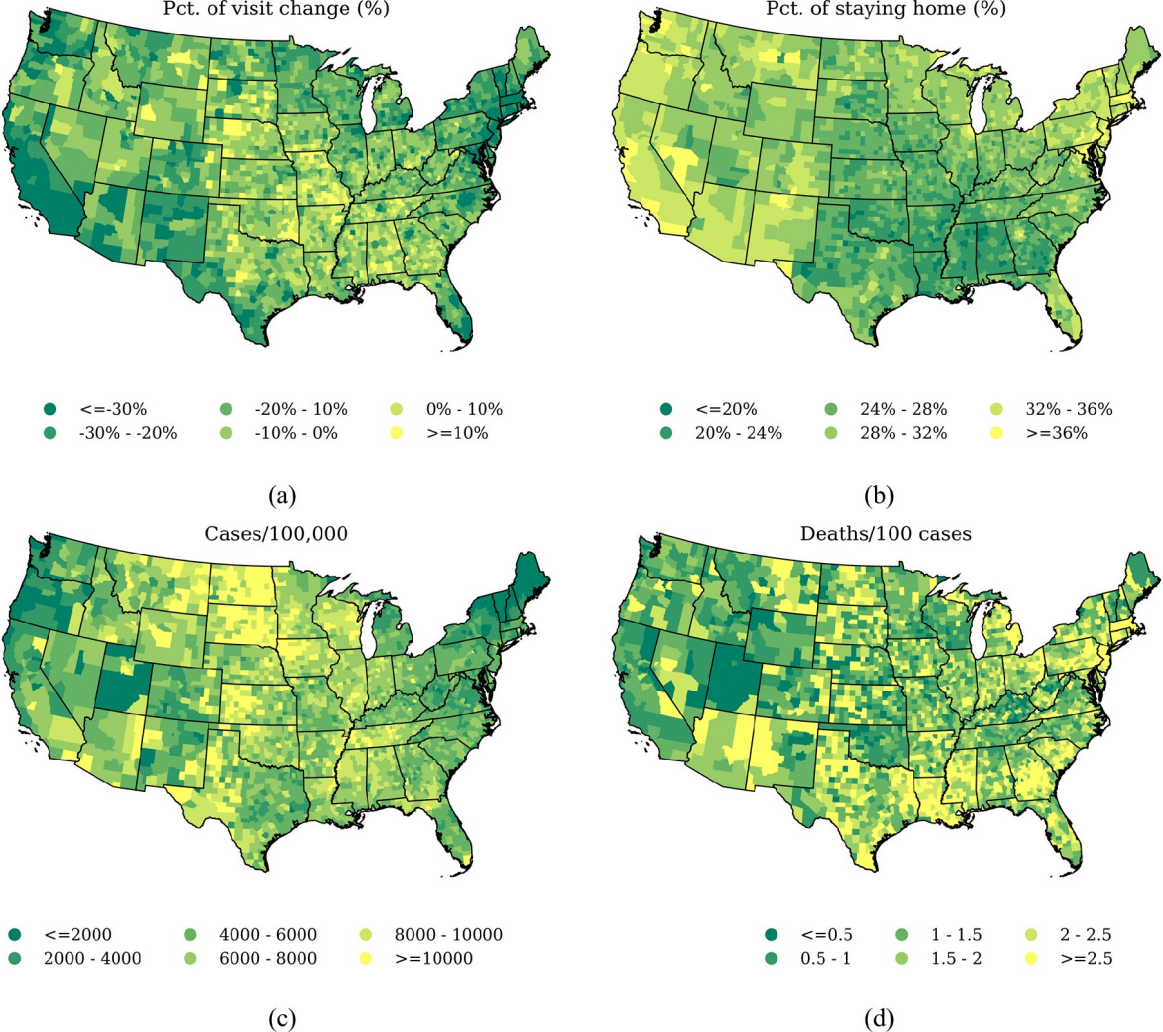
Spatial *d*istribution of *h*uman *m*obility and COVID-19 *h*ealth *o*utcomes in the *c*ontiguous US. Each panel represents one metric from (a) average staying home (%) from 1 March to 31 December 2020 compared to 2019 baseline, (b) average visit change (%) from 1 March to 31 December 2020, (c) cumulative cases/100,000 by the end of 2020, and (d) cumulative deaths/100 cases by the end of 2020.

We mapped the Spatial distributions of county-level human mobility and COVID-19 outcomes in the contiguous US in Fig 2. The two human mobility metrics showed a reversed pattern against each other, which is reasonable since more negative visit change (%) or more positive staying home (%) both imply more intensive compliance with stay-at-home orders. The two COVID-19 health metrics, however, did not present an intuitively consistent pattern. For example, counties with the highest deaths/100 cases were mainly concentrated in the South while counties with the highest cases/100,000 were highly represented in the Midwest. Such discrepancy indicates infection rate and case-fatality rate may be driven by different underlying factors. Comparing human mobility against COVID-19 health outcomes, we observed a moderate negative relationship between human mobility and cases/100,000, while such negative relationship was not visible in deaths/100 cases. Additionally, Fig 2 demonstrates pronounced evidence of intra-state clustering, either manifesting as the spatial concentration within states or the spatial exclusion among different states. Such high spatial dependence may be because non-pharmaceutical policies and epidemic outbreaks were always targeted at the state level, as well as the significant racial residential segregation in the US. The pronounced regional dependence suggests the need for control for state effects when specifying models.

**Fig 3:**
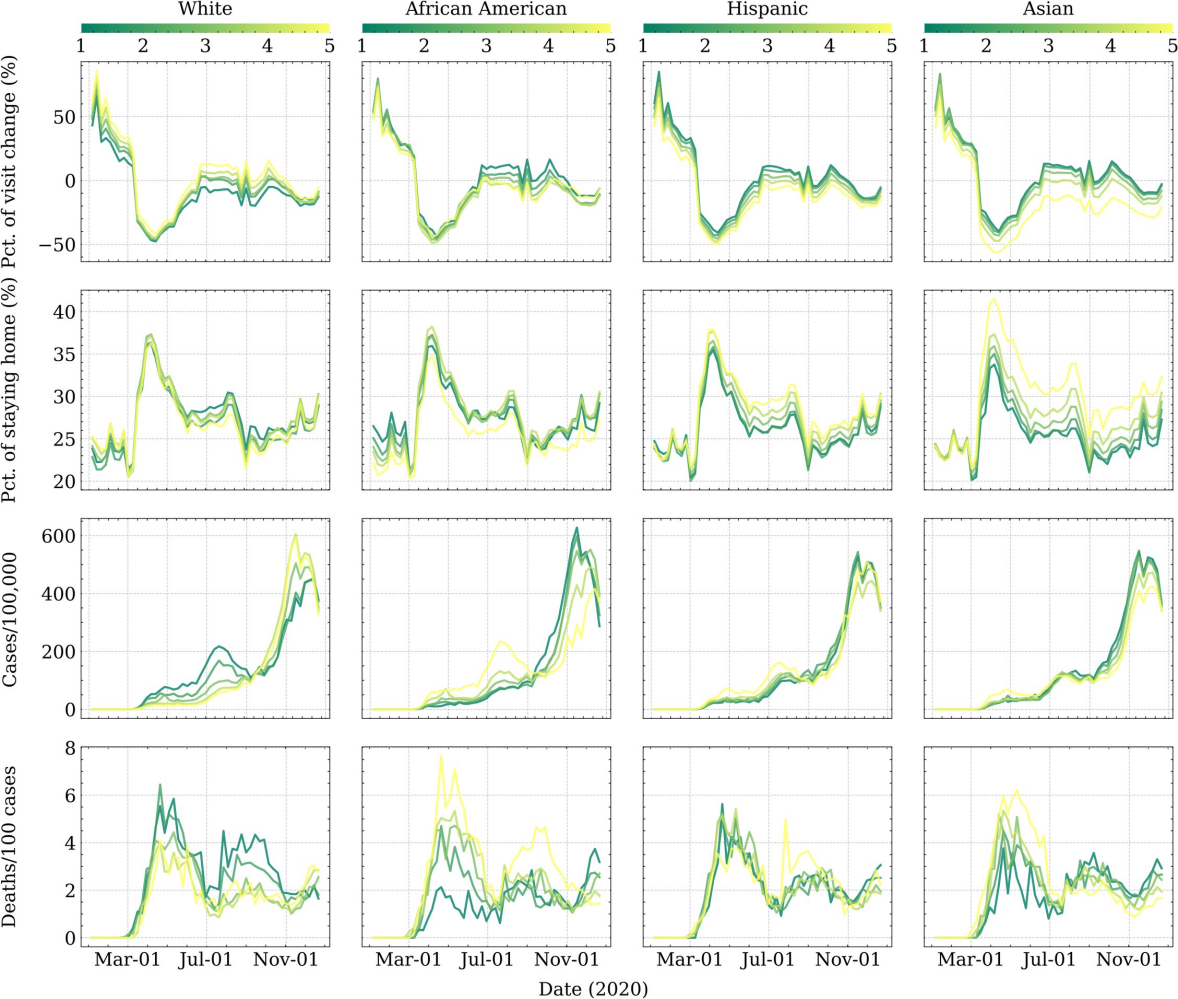
Temporal *e*volution of *h*uman *m*obility and COVID outcomes. Each row depicts a metric from (a) weekly percentage change in POI visits compared to 2019 baseline, (b) weekly percentage of residents staying home, (c) weekly new confirmed cases/100,000, and (d) weekly new deaths/100 cases. Each column represents a racial group, with each curve denoting one quintile. Sample comprises 3,108 contiguous US counties. Y-axis limits are shared by each row individually.

Temporal evolutions of four metrics stratified by racial make-up were delineated in Fig 3. Moving throughout COVID-19, we found POI visits plummeted steeply in mid-March until reaching their nadir during early April, followed by rapid recovery to the near-unperturbed baseline in June; afterward, racial differences in human mobility remained robust until the end of 2020. The shape of staying home percentage showed a similar inverse pattern compared with visit change. It sharply increased in March, then reaching the peak in April. It was followed by a precipitous fall to a plateau. Then, the second wave of fall occurred in August. It reached the nadir in September, which can be explained by the vanishing of the second-wave pandemic. Afterward, the staying home percentage gradually climbed back until the end of 2020.

We observed salient racial differences in two human mobility metrics in their temporal evolution patterns. During the pandemic, counties with a higher White population showed a worse stay-at-home ranking, i.e. less reduction in POI visits and lower staying home percentage. We also documented a reversal in the ordering of staying home by White: counties with more Whites went from most staying home before the pandemic to least staying home during the pandemic. African Americans showed some inconsistencies in two human mobility metrics. Counties in the highest African American quintile presented the greatest reduction in POI visits nonetheless the lowest staying home percentage, implying without controlling for other confounders, the currently observed gradients may not be robust. Asians and Hispanics presented similar patterns with each other. Counties with higher Asian or Hispanic populations indicated better compliance with stay-at-home orders, i.e. more reduction in POI visits and greater staying home percentage.

However, racial differences in two COVID-19 health outcome metrics explain the different patterns compared with human mobility. Broadly, the time-varying patterns of COVID-19 health outcomes were more complex, with more rises and fall, and experienced more changes regarding the orders of infection and fatality rates among racial groups. Specifically, White-leaning counties exhibited the lowest infection rate until September and became the highest afterward until early December when a precipitous fall occurred. Other racial groups, particularly African Americans, presented reversed patterns compared to Whites. Additionally, we noticed deaths/100 cases presented a lagged time-varying pattern compared with cases/100,000. For example, African American-dominating counties exhibited the highest deaths/100 cases until December, though their infection rate has decreased since September. White-leaning counties, on the contrary, presented the lowest deaths/100 cases throughout 2020 except December.

### 3.2. T-tests of four metrics between racial strata

To quantitatively compare the differences in the mean of the four metrics between racial strata, we conducted unpaired t-tests with the lowest quintile (hereafter Q1 for the lowest quintile and so on) as the reference (Table 2). Results were consistent with the findings in Fig 3. We found a decrease in compliance with stay-at-home orders with the increase of White populations. POI visits’ reduction in White Q1 counties was 12.45% greater (95% CI: 10.88, 14.02) than Q5, and residents staying home was 1.18% (95% CI: 0.70, 1.67) more. Nonetheless, we found the COVID-19 outcome presented an inverse gradient. Cases/100,000 and deaths/100 cases decreased with the increase of Whites. Counties in White Q1 experienced 931.03 (95% CI: 631.49, 1230.57) more cases/100,000 and 0.41 (95% CI: 0.28, 0.55) greater deaths/100 cases than Q5. Patterns in African Americans, however, were not cleanly monotonic. For example, counties in African American Q4 instead of Q5 showed the greatest drop-in visits and the highest staying home percentage. Also, a paradoxical relationship between staying home and visit change was observed in African American Q5 counties. Compared with Q1, African American Q5 counties presented more reduction in POI visits but lower staying home percentage. Such patterns underscore the need for a comprehensive model adjusting for other confounding factors.

**Table 2:** T-tests of difference in mean between quintiles of racial compositions (county level).

		Visit Change (%)		Staying home (%)		Cases/100,000		Deaths/100 Cases	
Racial groups	Quintile	Mean (SD)	Versus Q1 (95% CI)	Mean (SD)	Versus Q1 (95% CI)	Mean (SD)	Versus Q1 (95% CI)	Mean (SD)	Versus Q1 (95% CI)
**White**	Q1 (lowest)	-18.52 (16.21)	--	28.20 (5.22)	0.00*** (0.00, 0.00)	7080.72 (2840.00)	--	2.20 (1.26)	--
	Q2	-13.36 (14.41)	5.15*** (3.45, 6.86)	27.24 (4.88)	-0.97*** (-1.53, -0.40)	6270.02 (2626.08)	-810.70*** (-1115.09, -506.31)	1.88 (1.12)	-0.32*** (-0.45, -0.19)
	Q3	-13.05 (12.16)	5.47*** (3.88, 7.07)	28.16 (4.23)	-0.05 (-0.58, 0.48)	6216.55 (2971.46)	-864.17*** (-1187.51, -540.83)	1.56 (0.98)	-0.64*** (-0.77, -0.52)
	Q4	-10.25 (11.13)	8.27*** (6.72, 9.81)	27.91 (3.53)	-0.30 (-0.79, 0.20)	6622.61 (2848.74)	-458.11** (-774.67, -141.55)	1.58 (1.01)	-0.62*** (-0.75, -0.49)
	Q5 (highest)	-6.07 (11.69)	12.45*** (10.88, 14.02)	27.02 (3.18)	-1.18*** (-1.67, -0.70)	6149.66 (2536.47)	-931.05*** (-1230.59, -631.51)	1.80 (1.20)	-0.40*** (-0.54, -0.26)
**African American**	Q1 (lowest)	-7.05 (12.80)	--	27.55 (3.69)	0.00*** (0.00, 0.00)	6705.43 (3165.62)	--	1.70 (1.31)	--
	Q2	-11.19 (11.92)	-4.14*** (-5.51, -2.76)	28.15 (3.58)	0.60** (0.19, 1.00)	6315.98 (2862.50)	-389.46* (-725.31, -53.60)	1.64 (1.02)	-0.07 (-0.20, 0.06)
	Q3	-13.72 (13.43)	-6.67*** (-8.13, -5.21)	28.03 (4.15)	0.47* (0.04, 0.91)	6685.82 (2995.87)	-19.61 (-362.47, 323.24)	1.62 (1.01)	-0.09 (-0.22, 0.04)
	Q4	-16.32 (14.48)	-9.27*** (-10.79, -7.75)	28.62 (4.77)	1.07*** (0.59, 1.54)	6227.32 (2571.96)	-478.11** (-799.07, -157.14)	1.75 (0.98)	0.04 (-0.08, 0.17)
	Q5 (highest)	-12.98 (14.80)	-5.92*** (-7.46, -4.38)	26.18 (4.78)	-1.37*** (-1.85, -0.90)	6404.44 (2224.79)	-300.99. (-605.40, 3.41)	2.30 (1.21)	0.60*** (0.46, 0.74)
**Hispanic**	Q1 (lowest)	-6.79 (12.07)	--	26.65 (3.73)	0.00*** (0.00, 0.00)	6267.65 (2692.51)	--	1.95 (1.27)	--
	Q2	-8.50 (11.55)	-1.70* (-3.02, -0.39)	26.59 (3.57)	-0.06 (-0.47, 0.35)	6770.63 (2650.90)	502.98*** (205.64, 800.33)	1.83 (1.02)	-0.11. (-0.24, 0.01)
	Q3	-11.99 (12.19)	-5.20*** (-6.55, -3.85)	27.53 (3.97)	0.88*** (0.45, 1.31)	6588.29 (2773.74)	320.64* (16.56, 624.73)	1.74 (1.07)	-0.21** (-0.34, -0.08)
	Q4	-15.05 (13.82)	-8.26*** (-9.70, -6.81)	28.55 (4.35)	1.89*** (1.44, 2.35)	5929.68 (2721.62)	-337.97* (-639.25, -36.68)	1.65 (1.11)	-0.30*** (-0.43, -0.17)
	Q5 (highest)	-18.93 (15.74)	-12.14*** (-13.70, -10.58)	29.21 (5.10)	2.56*** (2.06, 3.05)	6782.99 (3005.03)	515.34** (197.94, 832.74)	1.85 (1.20)	-0.10 (-0.24, 0.04)
**Asian**	Q1 (lowest)	-5.08 (12.23)	--	25.49 (3.89)	0.00*** (0.00, 0.00)	6998.12 (3037.93)	--	1.97 (1.38)	--
	Q2	-5.93 (9.73)	-0.84 (-2.07, 0.39)	26.11 (3.14)	0.61** (0.22, 1.01)	6806.41 (2648.27)	-191.71 (-508.86, 125.44)	1.88 (1.10)	-0.09 (-0.23, 0.05)
	Q3	-9.17 (9.51)	-4.09*** (-5.31, -2.87)	27.14 (3.55)	1.64*** (1.23, 2.06)	6529.16 (2797.86)	-468.96** (-793.84, -144.07)	1.78 (1.06)	-0.19** (-0.33, -0.05)
	Q4	-14.65 (10.96)	-9.56*** (-10.85, -8.27)	28.52 (3.83)	3.02*** (2.59, 3.45)	6140.74 (2711.03)	-857.38*** (-1177.80, -536.97)	1.69 (0.98)	-0.28*** (-0.41, -0.15)
	Q5 (highest)	-26.42 (14.14)	-21.33*** (-22.80, -19.86)	31.27 (4.39)	5.77*** (5.31, 6.23)	5865.20 (2579.25)	-1132.92*** (-1446.42, -819.42)	1.69 (1.12)	-0.28*** (-0.42, -0.14)

Notes: Metrics are from (a) average percentage change in POI visits from 1 March to 31 December 2020, (b) average percentage of people staying home from 1 March to 31 December 2020, (c) cumulative number of COVID-19 cases/100,000 by the end of 2020, and (d) cumulative number of deaths/100 cases by the end of 2020. Q1 is for the lowest quintile and set as the reference group. Significance codes: 0 ‘***’ 0.001 ‘**’ 0.01 ‘*’ 0.05 ‘.’ 0.1 ‘‘ 1.

To quantitatively compare the differences in the mean value of the four metrics, we found a discrepant relationship between better compliance with stay-at-home orders against worse COVID-19 outcomes for Hispanics. Counties in Hispanic Q5 showed 12.14% (95% CI: 10.58, 13.70) higher reductions in POI visits and 2.56% (95% CI: 2.06, 3.05) more residents staying home than Q1. However, Hispanic Q5 counties experienced 515.34 (95% CI: 197.94, 832.74) greater cases/100,000. We found that positive feedback from stay-at-home orders to curb the spread of the virus among Asian communities. Among all racial groups, Asians exhibited the greatest difference in human mobility. Compared with Asian Q1 counties, Q5 exhibited 21.33% (95% CI: 19.86, 22.80) more reduction in POI visits, 5.77% (95% CI: 5.31, 6.23) more residents staying home, 1132.95 (95% CI: 819.45, 1446.44) fewer cases/100,000, and 0.28 (95% CI: 0.14, 0.42) lower deaths/100 cases.

For robustness check, we replicated the t-tests at the census block group (CBG) level on the two human mobility metrics. Only the human mobility metrics at the CBG level were checked since the CBG-level COVID-19 health outcomes were unavailable. It is worth mentioning since more than 20% of the CBGs have no Asian or African American residents, we defined the Q1 CBGs of the two races as the CBGs with zero residents in that race. Alternatively, 43.38% of CBGs belong to Asian Q1 and 27.82% of CBGs belong to African American Q1. Compared with county-level results, we found a similar gradient but greater racial differences (S1 Table). Specifically, POI visits’ reduction in White Q1 CBGs was 23.76% greater (95% CI: 23.41, 24.12) than Q5, and staying home percentage was 6.79% (95% CI: 6.71, 6.87) more; POI visits’ reduction in African American Q1 CBGs was 8.82% greater (95% CI: 8.48, 9.17) than Q5, and staying home percentage was 2.88% (95% CI: 2.80, 2.97) more; POI visits’ reduction in Hispanic Q1 CBGs was 19.04% greater (95% CI: 18.69, 19.04) than Q5, and staying home percentage was 4.96% (95% CI: 4.88, 5.04) more; POI visits’ reduction in Asian Q1 CBGs was 22.72% greater (95% CI: 22.41, 23.04) than Q5, and staying home percentage was 6.83% (95% CI: 6.74, 6.91) more. Such magnification substantiated racial disparities documented at the aggregated county level may be underestimated since portions of heterogeneity existing at a more localized spatial unit may be glossed over. We also tested other breakdown methods such as the Jenks natural break and found results broadly hold stable, which further proves the robustness of our findings (S2 Table).

### 3.3. Model fit measurements of SEMs

Preliminary analysis demonstrated pronounced racial disparities in human mobility and COVID-19 outcomes. We further examined the reliability of such disparities by controlling for confounders under an SEM framework and tested whether the differences in following stay-at-home orders among racial groups would influence their COVID-19 health outcomes through the mediation analysis. Four SEMs under different combinations of human mobility metrics and COVID-19 outcomes were fitted and the summary of model goodness-of-fit was reported in Table 3. All models showed reasonable model goodness-of-fit according to multiple SEM fit statistics including Chi-squared, Root means the square error of approximation (RMSEA), Comparative fit index (CFI), and SRMR (Standardized root mean squared residual). Rules of thumb guidelines are that CFI ≥ 0.95, TLI ≥ 0.95, RMSEA ≤ 0.06, and SRMR ≤ 0.08 represent a fitting model [43].

**Table 3:** Summary of SEM goodness-of-fit.

Mediator	Outcome	*χ* ^2^	AIC	SRMR	RMSEA (90% CI)	CFI
Visit change (%)	Cases/100,000	6.163	29045.801	0.000	0.019 (0.000, 0.040)	0.999
Staying home (%)	Cases/100,000	6.159	21491.535	0.000	0.018 (0.000, 0.039)	0.999
Visit change (%)	Deaths/100 cases	18.486	32879.085	0.001	0.041 (0.024, 0.060)	0.998
Staying home (%)	Deaths/100 cases	18.159	25286.520	0.001	0.034 (0.019, 0.050)	0.998

Since the four SEMs were not nested within each other, we compared among them via AIC. Broadly, the two SEMs with cases/100,000 as outcomes presented better goodness-of-fit (i.e. lower AIC) than the models with deaths/100 cases as outcomes. Also, under the same outcome, SEMs using staying home (%) as mediators exhibited better performance than SEMs using visit change (%) as mediators. For brevity, we only reported the results of models with staying home (%) as mediators in the following main text. Similar results with visit change (%) as mediators can be found in S3 and S4 Tables and S2 Fig.

### 3.4. Outputs of SEMs

Outputs of SEMs using staying home (%) as a mediator with both standardized and unstandardized estimations were reported in Table 4. After controlling for various confounders, we found a negative relationship between staying home and the COVID-19 infection rate. A 1% increase in staying home corresponded to 74.68 (95% CI: 37.23, 114.06) decrease in cases/100,000. However, we did not find a significant relationship between staying home (%) and deaths/100 cases.

**Table 4:** Output of structural equation models (mediator: Staying home (%)).

	Cases/100,000		Deaths/100 cases		Pct. of staying home (%)	
	Est. (95% CI) ^a^	Std. Est. ^b^	Est. (95% CI)	Std. Est.	Est. (95% CI)	Std. Est.
**Mediator**						
Staying home (%)	-74.68 (-114.06, -37.23) ***	-0.12	0.01 (-0.01, 0.03)	0.03	--	--
**Predictors**						
Asian	27.67 (-13.72, 78.51)	0.02	0.02 (-0.00, 0.04).	0.05	0.31 (0.25, 0.37) ***	0.17
African American	44.55 (32.40, 56.85) ***	0.23	0.01 (0.01, 0.02) ***	0.18	-0.02 (-0.03, 0.00) *	-0.05
Hispanic	75.48 (62.25, 86.71) ***	0.38	0.00 (-0.00, 0.01)	0.05	0.02 (0.01, 0.03) **	0.06
Other Minorities	59.30 (33.40, 78.68) ***	0.18	-0.00 (-0.01, 0.01)	-0.00	0.03 (0.01, 0.05) ***	0.06
**Control variables**						
Accommodation & Food	23.23 (-4.80, 53.33)	0.03	-0.02 (-0.04, -0.01) **	-0.07	0.00 (-0.03, 0.04)	0.00
Health Care	112.44 (71.61, 157.03) ***	0.14	0.02 (0.00, 0.03) *	0.06	0.00 (-0.04, 0.03)	0.00
Retail	9.21 (-26.87, 44.57)	0.01	0.01 (-0.01, 0.03)	0.02	-0.05 (-0.10, 0.01).	-0.03
Transportation	5.15 (-41.75, 43.46)	0.00	0.01 (-0.01, 0.04)	0.01	-0.03 (-0.11, 0.04)	-0.01
Education	24.73 (-6.64, 70.44)	0.03	-0.01 (-0.03, 0.01)	-0.02	-0.06 (-0.11, -0.02) **	-0.05
Manufacture	52.12 (33.58, 68.68) ***	0.13	0.00 (-0.01, 0.01)	0.02	-0.06 (-0.09, -0.04) ***	-0.10
GINI	3849.69 (756.74, 7954.65) *	0.05	-1.00 (-2.51, 0.90)	-0.03	-6.61 (-11.02, -2.65) **	-0.06
Administration	-35.00 (-89.36, 29.16)	-0.02	-0.07 (-0.10, -0.02) ***	-0.08	0.28 (0.18, 0.39) ***	0.09
Median Income	-45.05 (-154.38, 56.20)	-0.02	-0.07 (-0.12, -0.01) **	-0.09	0.12 (-0.01, 0.27).	0.04
Outlying	-495.77 (-673.89, -262.80) ***	-0.07	0.07 (-0.08, 0.15)	0.02	-1.19 (-1.43, -0.97) ***	-0.11
Rural	-148.69 (-367.24, 118.80)	-0.03	0.13 (0.02, 0.22) **	0.05	-1.31 (-1.56, -1.02) ***	-0.15
Without Insurance	-18.84 (-59.75, 6.43)	-0.03	0.03 (0.01, 0.04) ***	0.12	-0.01 (-0.06, 0.02)	-0.01
Male	185.81 (120.18, 265.59) ***	0.15	-0.04 (-0.06, -0.01) **	-0.07	-0.06 (-0.11, -0.01) *	-0.03
Age over 65	-67.51 (-92.79, -47.31) ***	-0.11	0.05 (0.03, 0.06) ***	0.19	0.11 (0.07, 0.14) ***	0.11
Population Density	164.43 (-138.25, 1599.56)	0.01	0.68 (0.43, 1.15) **	0.11	0.23 (-0.22, 2.14)	0.01
Democrats	-44.48 (-56.79, -34.25) ***	-0.24	-0.00 (-0.01, 0.00)	-0.02	0.08 (0.07, 0.10) ***	0.29

Note: a. Unstandardized estimation with 95% CI obtained through 1000 bootstraps; b. Standardized estimation (Z-scored). Significance codes: 0 ‘***’ 0.001 ‘**’ 0.01 ‘*’ 0.05 ‘.’ 0.1 ‘‘ 1. Similar results can be found in S3 Table with the visit change (%) setting as the mediator.

The predictors, i.e. the racial make-up, presented broadly consistent patterns with t-tests. During the pandemic, holding others constant and using Whites as reference, a county with 1% higher African American populations was associated with 0.02% (95% CI: 0.00, 0.03) lower staying home (%), 44.55 (95% CI: 32.40, 56.85) more cases/100,000, and 0.01 (95% CI: 0.01, 0.02) higher deaths/100 cases; a county with 1% higher Hispanic populations was associated with 0.02% (95% CI: 0.01, 0.03) greater staying home (%) and 75.48 (95% CI: 62.25, 86.71) more cases/100,000; a county with 1% higher Asian populations was associated with 0.31% (95% CI: 0.25, 0.37) higher staying home (%); a county with 1% higher other minorities populations was associated with 0.03% (95% CI: 0.01, 0.05) greater staying home (%) and 59.30 (95% CI: 33.40, 78.68) more cases/100,000. Altogether, SEMs suggested counties with higher Asian populations achieved the best in adhering to stay-at-home orders, followed by other minorities, Hispanics, Whites, and African Americans. The same order did not hold accordingly regarding COVID-19 health outcomes. Counties with more Whites and Asians reported the least cases/100,000, followed by African Americans, other minorities, and Hispanics. In addition, African American was the only group that showed statistically higher deaths/100 cases than White even after adjusting for extensive controls.

**Fig 4:**
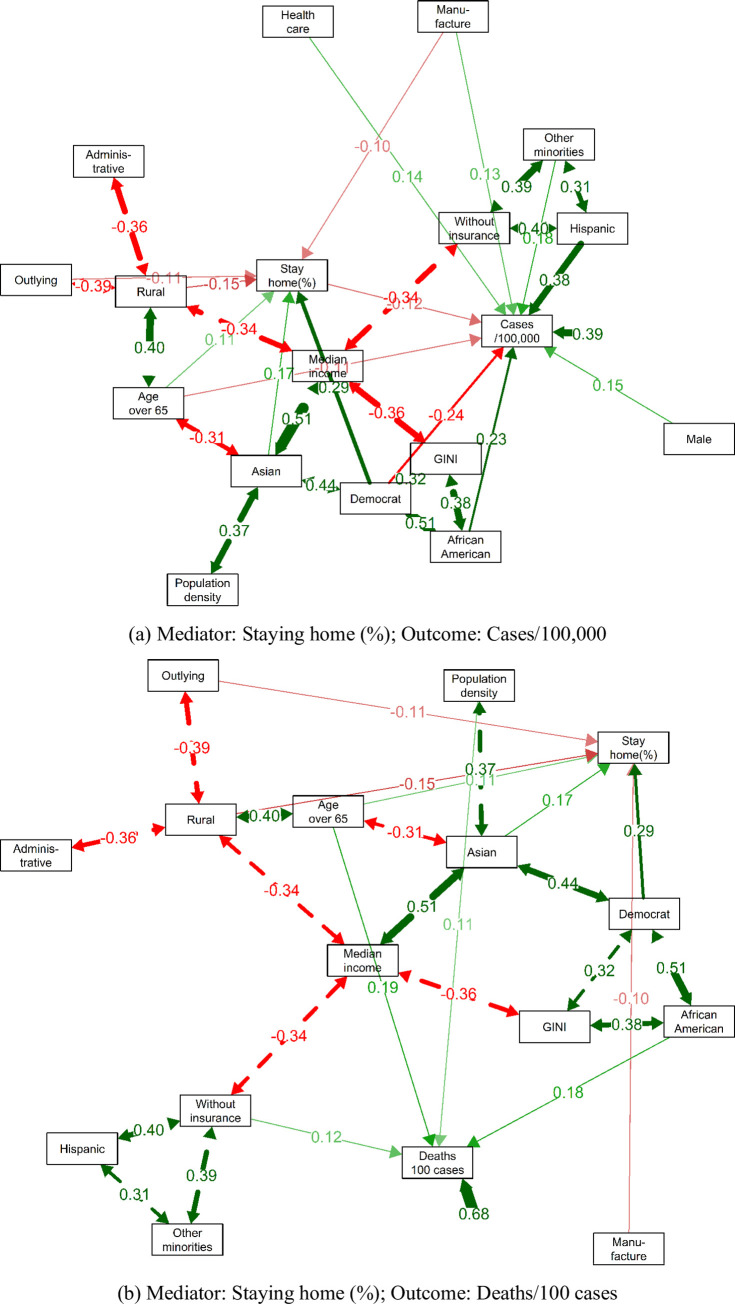
Standardized path diagrams. To clearly show the most important structure, we only depicted the paths with 1) P-value < 0.05, 2) standardized direct effect greater than 0.1, or 3) standardized covariate greater than 0.3. Double-headed dotted arrows represent the covariates between exogenous variables. Solid arrows represent the direct effect. The width of the arrow is proportional to the magnitude of standardized path coefficients. Red refers to a positive estimation while green represents a negative one. Similar diagrams can be found in S2 Fig with visit change (%) as mediator.

Control variables also accounted for the differences in human mobility and COVID-19 health outcomes. For the convenience of comparison across variables on different scales, we now move to the standardized estimation (see also the path diagrams in Fig 4). All interpretations now are based on the unit of variable’s standard deviation. The ranking of the standardized estimations in different models are as follows: 1) In regression with cases/100,000 as of the dependent variable, *Hispanics* presented the strongest positive relationship, followed by *African Americans*, *Other Minorities*, *Male*, *Health Care*, and *Manufacture*. *Democrats* presented the strongest negative relationship, followed by *Staying home (%)*, *Age over 65*, *and Outlying*. 2) In regression with deaths/100 cases as the dependent variable, *Age over 65* presented the strongest positive association, followed by *African Americans*, *Without Insurance*, and *Population Density*. *Median Income* presented the strongest negative association, followed by *Administration* and *Male*. 3) In regression with staying home (%) as the dependent variable, *Democrat* presented the strongest positive relationship, followed by *Asian*, *Age over 65*, and *Administration*. *Rural* presented the top negative relationship, followed by *Outlying* and *Manufacture*.

Some interplays among exogenous variables were also distinguishable in Fig 4. For example, we saw the triangle among *African American*, *GINI*, and *Democrat*; the entanglement among *Asian*, *Median income*, *Population density*, *Democrat*, and *Age over 65* with *Asian* as central; and the radial paths from *Median income* to *Asian*, *Rural*, *GINI*, and *Without insurance*, etc. Such strong pairwise correlations were consistent with the pairwise Pearson correlation shown in S1 Fig, and again necessitated the adjustment for confounding effects to disentangle the relationships between racial make-up and COVID-19 health outcomes.

It is worth mentioning that not all variables revealed significantly positive feedback from stay-at-home orders to mitigate the pandemic. For instance, *Hispanic* and *Other Minorities* were positively related to both compliance with stay-at-home orders and COVID-19 infection rate, while *Outlying* and *Rural* were negatively associated with both. Besides, various variables only presented significant associations in one model but not in another. For example, *Asian* was positively associated with compliance with stay-at-home orders but showed no significant associations with COVID-19 health outcomes. We also noticed stark differences between relationships in modeling cases/100,000 and deaths/100 cases. *Staying home (%)*, *Hispanic*, *Other Minorities*, *Manufacture*, *GINI*, *Outlying*, and *Democrats* were significantly associated with cases/100,000 but showed no significant associations with deaths/100 cases. On the other side, *Accommodation & Food*, *Administration*, *Median Income*, *Rural*, *Without Insurance*, and *Population Density* have significantly associated with deaths/100 cases but not with cases/100,000. Some variables even presented contradictory relationships. For example, counties with more elders were negatively related to case/100,000 but concurrently exhibited a considerably positive relationship with deaths/100 cases, implying although the infection ratio of elders was lower than other age groups, the mortality risk of those infected elders was higher.

### 3.5. Output of mediation analysis

The output of mediation analysis, including direct effects, indirect effects, and total effects with both standardized and unstandardized estimations, were recorded in Table 5. Direct effects are the same as the racial coefficients shown in Table 4. Mediation effects further revealed significant indirect links from racial make-up to cases/100,000 via stay-at-home orders, but such effects varied substantially among racial groups. Moreover, we found there was no significant indirect link from racial make-up to deaths/100 cases via stay-at-home orders. Alternatively, there was no statistically significant evidence to conclude that racial make-up can influence their case-fatality ratio through their stay-at-home behaviors.

**Table 5:** Output of mediation effects (mediator: Staying home (%)).

		Outcome					
		Cases/100,000			Deaths/100 cases		
**Predictor**		Est. (95% CI)	P-value	Std. Est.	Est. (95% CI)	P-value	Std. Est.
African American	Total effect	45.73*** (33.06, 57.95)	0.00	0.24	13.92*** (8.29, 19.34)	0.00	0.18
	Direct effect	44.55*** (32.40, 56.85)	0.00	0.23	14.05*** (8.43, 19.24)	0.00	0.18
	Indirect effect	1.19. (-0.05, 2.65)	0.06	0.01	-0.13 (-0.79, 0.17)	0.55	-0.00
Hispanic	Total effect	74.08*** (61.18, 84.80)	0.00	0.37	3.84. (-0.78, 8.66)	0.09	0.05
	Direct effect	75.48*** (62.25, 86.71)	0.00	0.38	3.69 (-1.08, 8.38)	0.11	0.05
	Indirect effect	-1.41* (-2.63, -0.37)	0.01	-0.01	0.16 (-0.24, 0.77)	0.47	0.00
Asian	Total effect	4.87 (-39.78, 48.88)	0.81	0.00	24.15* (1.05, 47.76)	0.04	0.05
	Direct effect	27.67 (-13.72, 78.51)	0.16	0.02	21.60. (-3.32, 44.54)	0.07	0.05
	Indirect effect	-22.80*** (-35.68, -11.42)	0.00	-0.02	2.55 (-3.41, 10.81)	0.44	0.01
Other minorities	Total effect	56.82*** (29.78, 74.55)	0.00	0.17	0.19 (-5.35, 6.80)	0.95	0.00
	Direct effect	59.30*** (33.40, 78.68)	0.00	0.18	-0.09 (-5.54, 6.77)	0.98	-0.00
	Indirect effect	-2.48* (-4.41, -1.01)	0.01	-0.01	0.28 (-0.40, 0.96)	0.42	0.00

Notes: 95% confidence intervals are in parentheses, which were obtained through 1000 bootstraps. White is set as the reference group. Both the standardized and unstandardized direct/indirect/total effects were reported. Significance codes: 0 ‘***’ 0.001 ‘**’ 0.01 ‘*’ 0.05 ‘.’ 0.1 ‘‘ 1. Similar results can be found in S4 Table when the visit change (%) was set as the mediator.

Counties with more Asians were a successful case to curb the spread of the virus via their salient compliance with stay-at-home orders. Compared with Whites, a county with 1% more Asians was associated with a 22.80 (95% CI: 11.42, 35.68) decrease in cases/100,000 through compliance with staying home. The magnitude of such indirect effect was very close to the corresponding direct effect. Adding them together led to an insignificant total effect from Asian to cases/100,000. As for Hispanics, although counties with higher Hispanic populations achieved some infection mitigations via stay-at-home orders, those mitigations were not as good as the direct effect. Compared with Whites, a county with 1% more Hispanics was associated with a 1.41 (95% CI: 0.37, 2.63) decrease in cases/100,000 through compliance with staying home. However, the direct effect from Hispanic to cases/100,000 was 75.48 (95% CI: 62.25, 84.80). Thus, the final total effect from Hispanic to cases/100,000 was still significantly positive. Similar patterns were documented regarding other minorities. Compared with Whites, a county with 1% more other minorities was associated with a 2.48 (95% CI: 1.01, 4.41) decrease in cases/100,000 through following stay-at-home orders, which was lower than their direct effect 59.30 (95% CI: 33.40, 78.68). Last, compared with Whites, counties with more African Americans were the only group that exacerbated their COVID-19 health outcomes, though not significant at .05 level, via their stay-at-home behaviors.

## 4. Discussion, policy implication, and conclusion

This work leveraged large-scale human movement data and COVID-19 records in 2020 to examine county-level racial differences in human mobility and COVID-19 health outcomes across the contiguous US. After adjusting for extensive confounders, such as state effects, socioeconomics, demographics, occupation, and partisanship, we found racial make-up was more strongly associated with human mobility and COVID-19 infection and death rates than numerous other factors. Counties with more Asians exhibited the greatest compliance with stay-at-home orders, both in terms of reducing their POI visits and increasing their staying home percentage, followed by other minorities, Hispanics, Whites, and African Americans. However, racial differences in COVID-19 health outcomes present discrepant gradients. Counties with more Whites and Asians experienced the lowest infection rate, followed by African Americans, other minorities, and Hispanics. Such paradoxicality, i.e. counties with more racial compositions performing better stay-at-home behaviors do not always experience lower infection and death rates, indicates the considerably unequal risk of COVID-19 outcomes experienced by different racial compositions cannot be simply explained by their degree of staying home.

Preliminary analysis via t-test uncovered stark racial disparities in human mobility and COVID-19 health outcomes. However, such disparities were not monotonous among racial quintiles and cannot provide solid evidence regarding the racial gradient. For example, although t-tests showed counties with higher White populations presented worse compliance with stay-at-home orders, we still cannot conclude whether counties with more White populations did worse than African Americans or not. This partially helps explain why in current literature it is inconclusive on racial differences in following stay-at-home orders. Researchers with the conclusion of African American-leaning regions did better in following stay-at-home orders [32, 33] were mostly based on preliminary analysis or partially-control models without full adjustment of underlying confounders.

We further linked racial differences in human mobility to COVID health outcomes via mediation analyses. We did not find enough evidence to claim a significant link between the case-fatality ratio and racial groups via stay-at-home orders. It is plausible as staying home may curb the transmission of the virus but could not allay the fatality risk of those confirmed cases. Results also corroborated that staying home did have non-negligible effects on reducing infection rate [5], but the effects varied substantially among racial groups. Counties with larger groups of Asian Americans appear to be the only case to successfully curtail COVID-19 infection via staying home, while other racial groups failed to suppress the epidemic via staying home either due to the slight indirect effect or the salient direct effect. We argue this may be because the racial make-up, socioeconomics, health care resources, and pre-existing health issues are closely intertwined and hard to fully disentangle. For example, *Asian* is highly positively related to variables indicating high socioeconomic status such as *Median Income* (Pearson = 0.508) while *Hispanic* is positively associated with *Without Insurance* (Pearson = 0.402). Meanwhile, previous studies [10, 25, 44] claimed that racial and ethnic minorities, particularly African Americans and Hispanics, have, on average, systematically lower socioeconomic positions than Whites. And it has disproportionately resided in low-income communities due to an entrenched discriminatory housing policy.

Our work highlights racial disparities in the US are now irrefutably far-reaching and pernicious enough to threaten the health and safety of residents during the COVID-19 pandemic. A significant and positive relationship was observed between the case-fatality ratio and African Americans even after adjusting for extensive controls, which was consistent with prior studies [23, 24, 31]. Given that COVID-19 does not intrinsically discriminate across racial/ethnic groups, reasons for such differences deserved more investigation. The deeply embedded systematic and structural racism may be one explanation. Prior studies claimed that even those African Americans who possess high income and outstanding health insurance coverage still frequently received inferior care due to implicit bias among healthcare providers [29]. Other reasons might include unobserved disadvantaged socioeconomic statuses such as food and housing insecurity and crowded working and living conditions. Also, reasons that including an uneven distribution of scarce testing and hospital resources, high concentration of workers in essential services with a high degree of exposure to human interaction, and the confluence of other underlying comorbidities such as hypertension, obesity, renal disease, heart disease, and diabetes, may all potentially account for such high case-fatality ratio [23, 26, 27, 31, 44, 45].

The standardized SEMs substantiated that control variables also accounted for the differences in human mobility and COVID-19 health outcomes. Similar to previous studies [21, 22], we found the most significant effects on human mobility were from partisanship. In addition, we found counties with higher median household income, fewer manufacturing industries, or located in central regions, adhered better to stay-at-home orders, supporting findings in mounting previous studies that higher socioeconomic position affords greater opportunity to adjust their travel behaviors [2, 9, 10, 19, 21, 22, 26, 42, 46, 47]. However, not all variables indicating high socioeconomic status simultaneously exhibited negative relationships with COVID-19 outcomes. Some variables may exhibit paradoxical relationships, while some may only show significance on one side. Such finding again substantiated that stay-at-home orders may not always be effective in containing the spread of the virus when communities were under specific socioeconomic status. Additionally, our work also uncovered that infection rate and the case-fatality rate are substantially different, both in terms of temporal evolutions and underlying determinants. We documented a lagged effect between infection rate and the case-fatality rate through their temporal evolution, which is plausible since there should exist a time gap from a patient’s infection to death. The difference in underlying factors, however, highlighted the importance of examining infection and death rates separately in future research.

Findings provide a glimpse of the impact of various potential determinants, such as racial disparities, socioeconomics, demographics, occupation, and partisanship on human mobility and COVID-19 outcome. It also inspires new avenues for more effective and equitable policies for preventing the possible emergence of future epidemic crises. When it comes to the effectiveness in curbing the spread of the virus, stay-at-home order is suggested as a regular practice until most populations are successfully vaccinated towards herd immunity. Meanwhile, governments should focus more on complementary policies that could facilitate the implementation of non-pharmaceutical interventions among socially vulnerable populations to help protect the most vulnerable. Such services could also consider: selectively loosening the stay-at-home orders, introducing national educational campaigns to increase self-protective awareness, making unemployment insurance and paid sick leave more readily available, instituting additional disinfection procedures in “essential” workplaces, public space, and transit lines seeing sustained flows, and allocating protective gear, tests, vaccinations, and relief payment with higher priority to those with higher risks of exposure or at increased risks of severe illness, etc.

Several limitations are recognized and deserve further research. First, associations revealed in our models are not intended for drawing causal inferences. The estimations may be biased downwards due to the reverse causality between COVID-19 health outcomes and human mobility. For example, counties with higher infection rates may have greater panic effects on citizens to curtailing their mobility; meanwhile, the decrease in human mobility will simultaneously flatten the infection curve. Second, all analyses were conducted at an aggregate level instead of the individual level; hence, conclusions might not be generalizable or reflective of the actual individual behavior. Third, our mobility data only represents the behavior of residents with mobile devices and opt-in to location tracking and does not collect data from every device at regular intervals throughout the day, which may render spatiotemporal sample biases [48]. Fourth, the number of COVID-19 cases and deaths might not accurately reflect the real situation due to the variability in access to testing resources and recommendations for who should be tested. Last, this study mainly focuses on the cross-sectional modeling on an average level, which ignores the potential time-varying patterns in relationships among human mobility, COVID-19 health outcomes, and controls, further analysis should consider incorporating such temporal dynamics.

## Supporting information

S1 Fig Pairwise pearson correlation heatmap. (DOCX)

S2 Fig Standardized path diagrams. (DOCX)

S1 Table T-tests of difference in mean between quintiles of four racial groups (CBG level). (DOCX)

S2 Table T-tests of difference in mean between quintiles of four racial groups (county level, Jenks natural breaks). (DOCX)

S3 Table Output of regression models (mediator: Visit change (%)). (DOCX)

S4 Table Output of mediation effects (mediator: Visit change (%)). (DOCX)
