# Supplementary material for: Do racial and ethnic disparities in following stay-at-home orders influence COVID-19 health outcomes? A mediation analysis approach
Source: PLoS One. 2021 Nov 11;16(11):e0259803. doi: 10.1371/journal.pone.0259803 (PMC8584966; doi:10.1371/journal.pone.0259803)
Supplement: S1 Fig — (DOCX) [file pone.0259803.s001.docx]

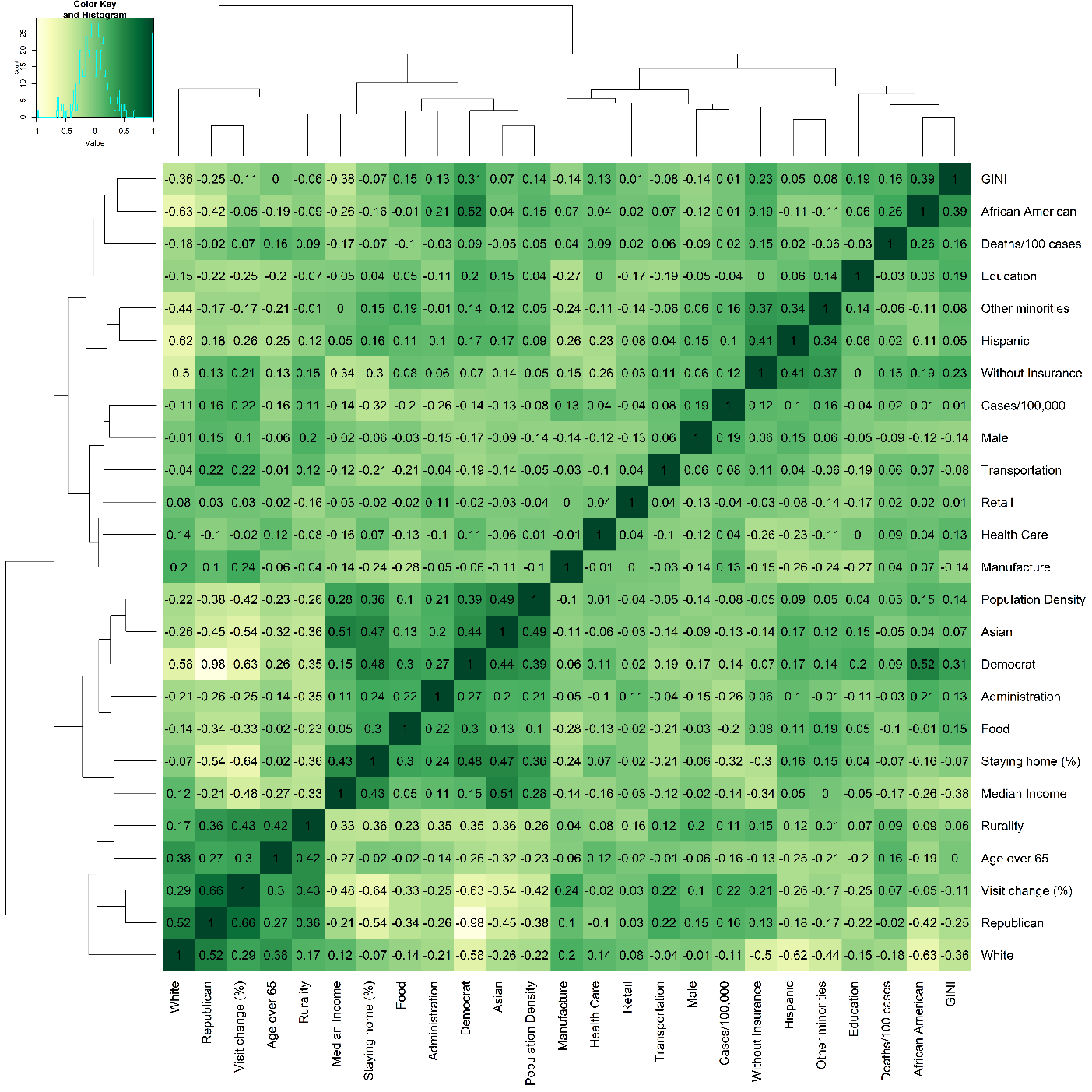


**Figure S1. Pairwise Pearson Correlation Heatmap**. Rows and columns are reordered by the dendrogram.
