# Supplementary material for: Do racial and ethnic disparities in following stay-at-home orders influence COVID-19 health outcomes? A mediation analysis approach
Source: PLoS One. 2021 Nov 11;16(11):e0259803. doi: 10.1371/journal.pone.0259803 (PMC8584966; doi:10.1371/journal.pone.0259803)
Supplement: S2 Fig — (DOCX) [file pone.0259803.s002.docx]

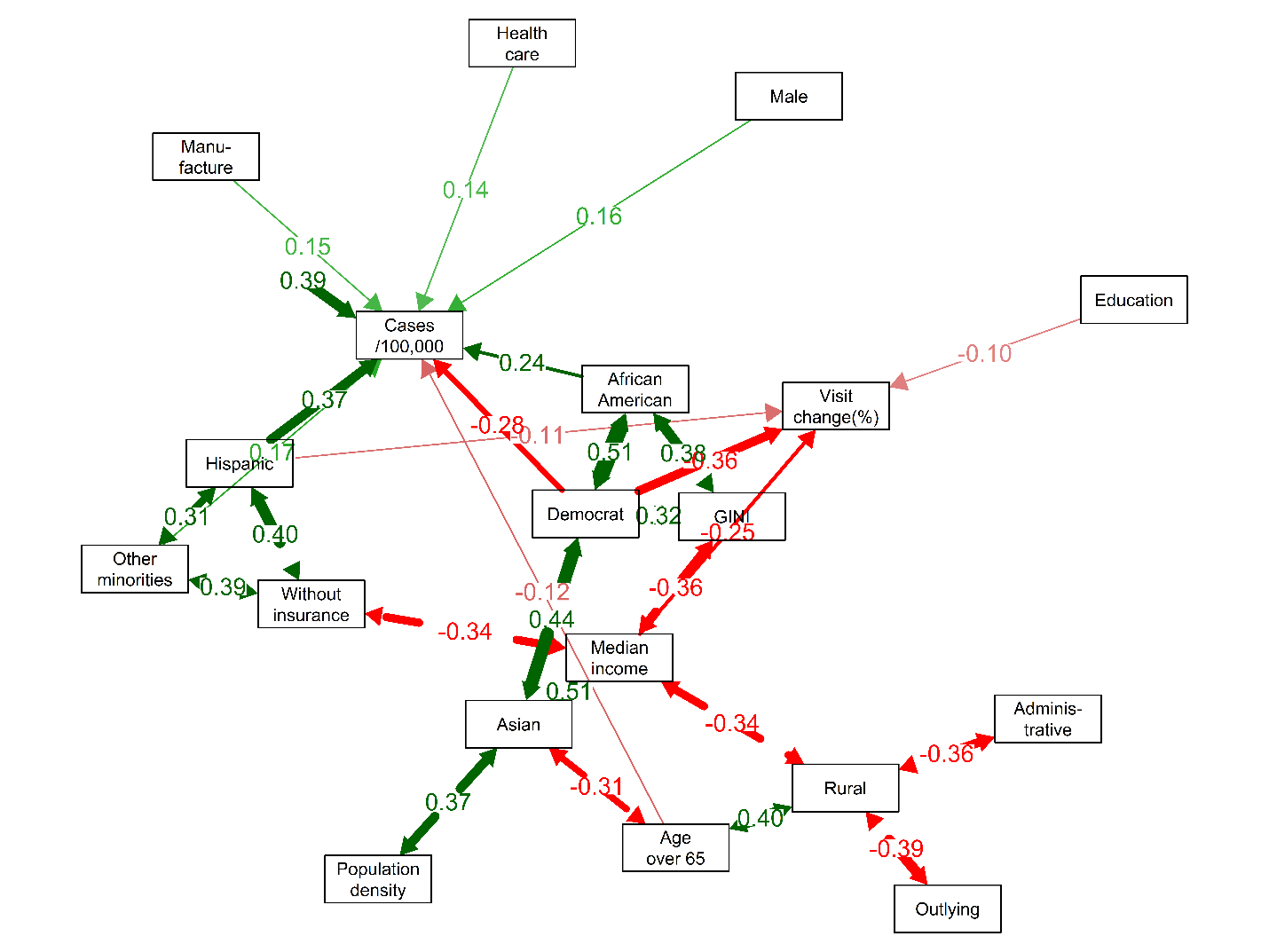


(a) Mediator: Visit change (%); Outcome: Cases/100,000


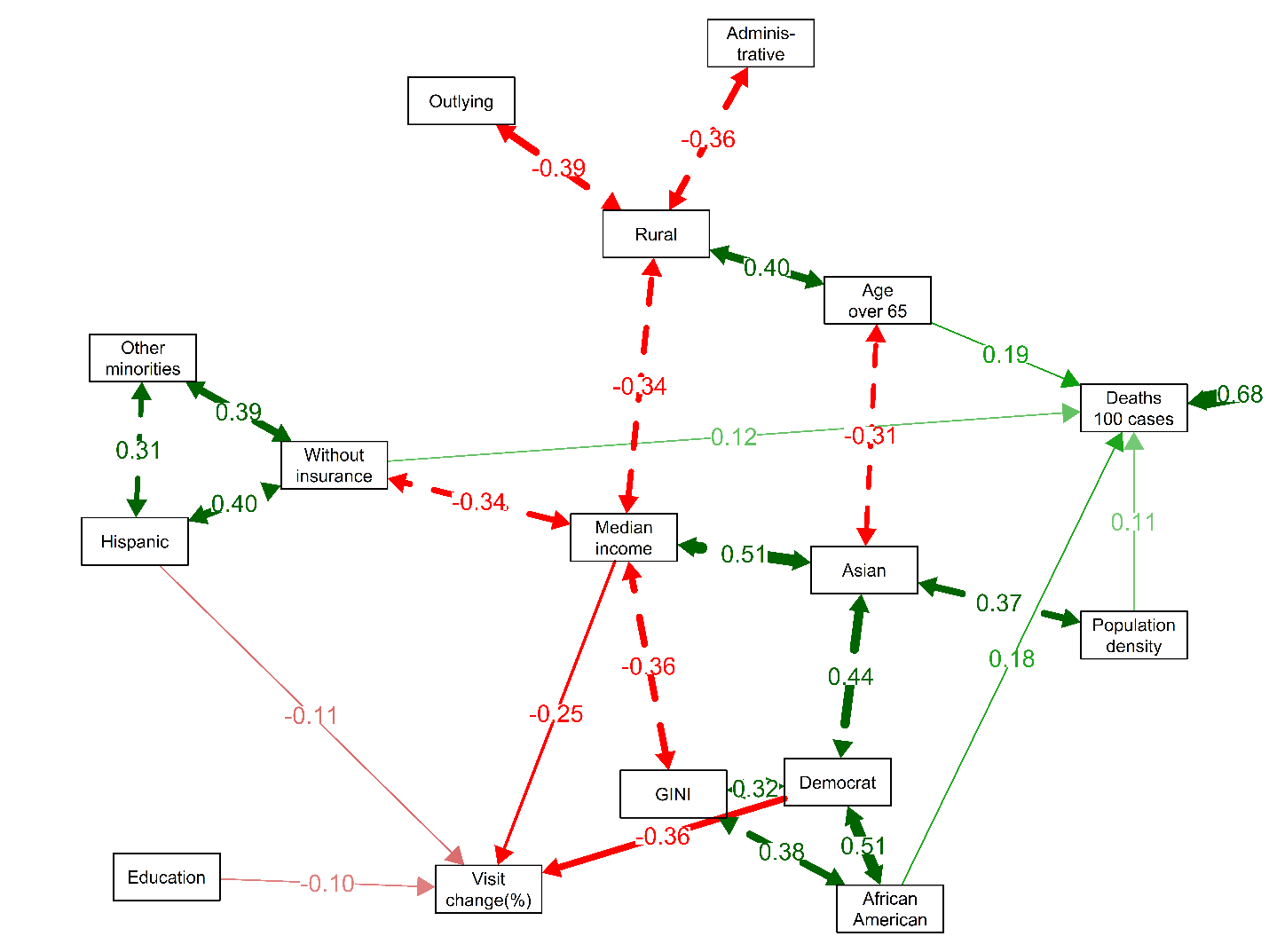


(b) Mediator: Visit change (%); Outcome: Deaths/100 cases

**Figure S2. Standardized Path Diagrams**. This figure is analogous to Figure 5 in the main text except the mediator is visit change (%).
