# Supplementary material for: Do racial and ethnic disparities in following stay-at-home orders influence COVID-19 health outcomes? A mediation analysis approach
Source: PLoS One. 2021 Nov 11;16(11):e0259803. doi: 10.1371/journal.pone.0259803 (PMC8584966; doi:10.1371/journal.pone.0259803)
Supplement: S1 Table — (DOCX) [file pone.0259803.s003.docx]

**Table S1. T-tests of Difference in Mean between Quintiles of Four Racial Groups (CBG level)**

|  |  | **Visits Change (%)** | | **Staying home (%)** | |
| --- | --- | --- | --- | --- | --- |
| **Racial groups** | **Quintile** | **Mean (St.d.)** | **Versus Q1**  **(95% CI)** | **Mean (St.d.)** | **Versus Q1**  **(95% CI)** |
| **White** | Q1  (lowest) | -34.28 (20.79) | -- | 34.95 (6.21) | 0.00*** (0.00, 0.00) |
|  | Q2 | -28.77 (24.70) | 5.51*** (5.20, 5.82) | 32.88 (6.61) | -2.07*** (-2.16, -1.99) |
|  | Q3 | -24.84 (26.77) | 9.44*** (9.12, 9.76) | 31.11 (6.42) | -3.84*** (-3.93, -3.76) |
|  | Q4 | -19.21 (27.69) | 15.07*** (14.74, 15.40) | 29.89 (5.96) | -5.06*** (-5.15, -4.98) |
|  | Q5 (highest) | -10.52 (31.04) | 23.76*** (23.41, 24.12) | 28.16 (5.48) | -6.79*** (-6.87, -6.71) |
| **African American** | Q1  (lowest) | -17.80 (31.37) | -- | 29.94 (6.24) | 0.00*** (0.00, 0.00) |
|  | Q2 | -22.59 (27.29) | -4.79*** (-5.16, -4.41) | 31.12 (6.34) | 1.18*** (1.10, 1.26) |
|  | Q3 | -26.71 (26.38) | -8.91*** (-9.28, -8.55) | 31.87 (6.67) | 1.93*** (1.84, 2.01) |
|  | Q4 | -26.95 (25.29) | -9.16*** (-9.52, -8.80) | 32.01 (6.72) | 2.07*** (1.98, 2.15) |
|  | Q5 (highest) | -26.62 (23.58) | -8.82*** (-9.17, -8.48) | 32.82 (6.67) | 2.88*** (2.80, 2.97) |
| **Hispanic** | Q1  (lowest) | -13.96 (30.62) | -- | 28.87 (6.19) | 0.00*** (0.00, 0.00) |
|  | Q2 | -19.10 (28.53) | -5.15*** (-5.55, -4.75) | 30.12 (6.42) | 1.25*** (1.16, 1.33) |
|  | Q3 | -24.02 (27.19) | -10.07*** (-10.46, -9.68) | 31.42 (6.65) | 2.54*** (2.46, 2.63) |
|  | Q4 | -27.52 (25.66) | -13.57*** (-13.95, -13.19) | 32.73 (6.51) | 3.86*** (3.77, 3.94) |
|  | Q5 (highest) | -33.00 (21.32) | -19.04*** (-19.40, -18.69) | 33.83 (5.91) | 4.96*** (4.88, 5.04) |
| **Asian** | Q1  (lowest) | -16.10 (28.71) | -- | 29.45 (6.10) | 0.00*** (0.00, 0.00) |
|  | Q2 | -20.28 (26.12) | -4.19*** (-4.54, -3.84) | 30.51 (5.98) | 1.06*** (0.98, 1.14) |
|  | Q3 | -26.08 (25.36) | -9.99*** (-10.33, -9.64) | 31.75 (6.02) | 2.30*** (2.22, 2.38) |
|  | Q4 | -31.69 (23.82) | -15.59*** (-15.92, -15.27) | 32.99 (6.07) | 3.53*** (3.45, 3.61) |
|  | Q5 (highest) | -38.82 (22.20) | -22.72*** (-23.04, -22.41) | 36.28 (6.68) | 6.83*** (6.74, 6.91) |

Notes: This table is analogous to Table 2 human mobility part in the main text except the spatial unit is CBG level.
