# Supplementary material for: Do racial and ethnic disparities in following stay-at-home orders influence COVID-19 health outcomes? A mediation analysis approach
Source: PLoS One. 2021 Nov 11;16(11):e0259803. doi: 10.1371/journal.pone.0259803 (PMC8584966; doi:10.1371/journal.pone.0259803)
Supplement: S2 Table — (DOCX) [file pone.0259803.s004.docx]

**Table S2. T-tests of difference in Mean between Quintiles of Racial Compositions (county level, Jenks natural breaks)**

|  |  | **Visit Change (%)** | | **Staying home (%)** | | **Cases/100,000** | | **Deaths/100 Cases** | |
| --- | --- | --- | --- | --- | --- | --- | --- | --- | --- |
| **Racial groups** | **Break** | **Mean (SD)** | **Versus Q1**  **(95% CI)** | **Mean (SD)** | **Versus Q1**  **(95% CI)** | **Mean**  **(SD)** | **Versus Q1**  **(95% CI)** | **Mean (SD)** | **Versus Q1**  **(95% CI)** |
| **White** | Q1  (lowest) | -24.38 (15.04) | -- | 29.68 (5.24) | 0.00*** (0.00, 0.00) | 8647.74 (3372.12) | -- | 2.51 (1.53) | -- |
|  | Q2 | -20.13 (16.16) | 4.25* (0.69, 7.81) | 28.74 (5.41) | -0.94  (-2.17, 0.29) | 6872.82 (2644.98) | -1774.92*** (-2519.86, -1029.98) | 2.17 (1.24) | -0.34. (-0.68, 0.00) |
|  | Q3 | -13.76 (15.15) | 10.62*** (7.34, 13.89) | 26.99 (4.84) | -2.70*** (-3.82, -1.57) | 6594.41 (2746.83) | -2053.33*** (-2768.94, -1337.73) | 1.97 (1.13) | -0.53** (-0.86, -0.21) |
|  | Q4 | -13.90 (13.17) | 10.47*** (7.30, 13.65) | 28.04 (4.64) | -1.64** (-2.75, -0.54) | 6029.51 (2693.01) | -2618.23*** (-3324.51, -1911.95) | 1.68 (1.06) | -0.82*** (-1.14, -0.51) |
|  | Q5 (highest) | -8.41 (11.51) | 15.97*** (12.88, 19.06) | 27.47 (3.40) | -2.21*** (-3.28, -1.14) | 6413.68 (2759.32) | -2234.05*** (-2928.23, -1539.88) | 1.67 (1.10) | -0.83*** (-1.15, -0.52) |
| **African American** | Q1  (lowest) | -11.33 (12.93) | -- | 28.01 (3.84) | 0.00*** (0.00, 0.00) | 6500.88 (2954.12) | -- | 1.67 (1.08) | -- |
|  | Q2 | -16.67 (14.99) | -5.34*** (-6.79, -3.88) | 28.30 (4.96) | 0.29  (-0.19, 0.76) | 6219.78 (2420.09) | -281.11* (-533.23, -28.99) | 1.79 (0.93) | 0.12* (0.03, 0.22) |
|  | Q3 | -13.82 (15.19) | -2.49* (-4.39, -0.60) | 26.46 (5.10) | -1.56*** (-2.19, -0.93) | 6148.22 (2306.77) | -352.67* (-656.36, -48.97) | 2.04 (1.09) | 0.37*** (0.23, 0.51) |
|  | Q4 | -11.11 (14.54) | 0.22  (-1.99, 2.44) | 25.60 (4.39) | -2.41*** (-3.08, -1.74) | 6411.00 (1865.41) | -89.89  (-393.73, 213.95) | 2.53 (1.18) | 0.86*** (0.68, 1.04) |
|  | Q5 (highest) | -12.94 (13.49) | -1.61  (-4.49, 1.27) | 26.15 (4.06) | -1.87*** (-2.73, -1.00) | 7313.84 (2242.55) | 812.95** (326.16, 1299.75) | 2.83 (1.45) | 1.17*** (0.86, 1.48) |
| **Hispanic** | Q1  (lowest) | -9.89 (12.29) | -- | 27.10 (3.85) | 0.00*** (0.00, 0.00) | 6490.60 (2726.17) | -- | 1.82 (1.12) | -- |
|  | Q2 | -16.56 (15.10) | -6.67*** (-8.05, -5.30) | 29.14 (4.79) | 2.04*** (1.60, 2.47) | 5896.99 (2749.45) | -593.60*** (-852.06, -335.15) | 1.68 (1.12) | -0.14* (-0.24, -0.03) |
|  | Q3 | -18.44 (16.54) | -8.55*** (-10.77, -6.32) | 29.19 (5.08) | 2.09*** (1.40, 2.77) | 6771.35 (3111.39) | 280.76  (-141.96, 703.47) | 1.81 (1.21) | -0.01  (-0.17, 0.16) |
|  | Q4 | -20.47 (14.93) | -10.58*** (-13.24, -7.92) | 28.86 (5.09) | 1.76*** (0.85, 2.66) | 7540.58 (2903.88) | 1049.98*** (529.46, 1570.50) | 1.87 (1.17) | 0.06  (-0.15, 0.27) |
|  | Q5 (highest) | -25.72 (9.24) | -15.83*** (-18.96, -12.71) | 29.23 (5.06) | 2.13* (0.44, 3.82) | 8267.13 (2585.23) | 1776.54*** (907.52, 2645.55) | 2.45 (1.53) | 0.64* (0.13, 1.15) |
| **Asian** | Q1  (lowest) | -9.12 (10.79) | -- | 26.94 (3.68) | 0.00*** (0.00, 0.00) | 6577.18 (2741.40) | -- | 1.83 (1.08) | -- |
|  | Q2 | -22.03 (11.81) | -12.91*** (-14.08, -11.74) | 30.04 (3.91) | 3.10*** (2.71, 3.49) | 6104.74 (2609.56) | -472.45*** (-737.31, -207.58) | 1.65 (1.01) | -0.18*** (-0.28, -0.07) |
|  | Q3 | -35.47 (13.69) | -26.36*** (-28.74, -23.98) | 33.47 (3.53) | 6.53*** (5.91, 7.15) | 5491.24 (2590.82) | -1085.94*** (-1542.74, -629.15) | 1.65 (1.21) | -0.17  (-0.38, 0.04) |
|  | Q4 | -43.19 (9.00) | -34.07*** (-37.29, -30.85) | 37.10 (2.85) | 10.17*** (9.15, 11.19) | 4524.30 (1413.09) | -2052.89*** (-2565.61, -1540.16) | 2.17 (1.65) | 0.35  (-0.24, 0.93) |
|  | Q5 (highest) | -56.11 (8.28) | -46.99*** (-55.67, -38.31) | 41.75 (1.43) | 14.82*** (13.31, 16.32) | 3797.94 (1329.52) | -2779.24** (-4173.00, -1385.48) | 2.32 (2.18) | 0.50  (-1.79, 2.78) |

Notes: This table is analogous to Table 2 in the main text except the breakdown method is Jenks natural break
