# Supplementary material for: Do racial and ethnic disparities in following stay-at-home orders influence COVID-19 health outcomes? A mediation analysis approach
Source: PLoS One. 2021 Nov 11;16(11):e0259803. doi: 10.1371/journal.pone.0259803 (PMC8584966; doi:10.1371/journal.pone.0259803)
Supplement: S3 Table — (DOCX) [file pone.0259803.s005.docx]

**Table S3. Output of Regression Models (Mediator: Visit change (%))**

|  | **Cases/100,000** | | **Deaths/100 cases** | | | **Visit change (%)** | |
| --- | --- | --- | --- | --- | --- | --- | --- |
|  | **Est. (95% CI)** | **Std. Est.** | **Est. (95% CI)** | **Std. Est.** | **Est. (95% CI)** | | **Std. Est.** |
| **Mediator** |  |  |  |  |  | |  |
| Visit change (%) | -5.54 (-16.68, 5.51) | -0.03 | 0.00 (-0.00, 0.01) | 0.03 |  | |  |
| **Predictors** |  |  |  |  |  | |  |
| Asian | 2.81 (-29.30, 41.95) | 0.00 | 0.02 (-0.00, 0.04) * | 0.05 | -0.37 (-0.52, -0.18) *** | | -0.06 |
| African American | 46.05 (33.70, 57.55) *** | 0.24 | 0.01 (0.01, 0.02) *** | 0.18 | 0.06 (0.01, 0.11) * | | 0.06 |
| Hispanic | 73.45 (62.90, 87.53) *** | 0.37 | 0.00 (-0.00, 0.01) | 0.05 | -0.11 (-0.15, -0.08) *** | | -0.11 |
| Other minorities | 56.55 (36.75, 73.07) *** | 0.17 | 0.00 (-0.01, 0.00) | 0.00 | -0.05 (-0.13, 0.02) | | -0.03 |
| **Control variables** | | | | | | | |
| Accommodation & Food | 21.66 (-4.88, 45.42) | 0.03 | -0.02 (-0.04, -0.01) ** | -0.07 | -0.28 (-0.43, -0.14) *** | | -0.07 |
| Health Care | 112.70 (78.28, 144.18) *** | 0.14 | 0.02 (0.00, 0.04) * | 0.06 | 0.05 (-0.09, 0.15) | | 0.01 |
| Retail | 12.21 (-12.52, 43.00) | 0.01 | 0.01 (-0.01, 0.03) | 0.02 | -0.07 (-0.25, 0.15) | | -0.01 |
| Transportation | 8.01 (-31.42, 48.48) | 0.01 | 0.01 (-0.02, 0.03) | 0.01 | 0.11 (-0.11, 0.34) | | 0.02 |
| Education | 27.18 (-6.06, 59.96). | 0.03 | -0.01 (-0.03, 0.01) | -0.02 | -0.43 (-0.59, -0.28) *** | | -0.10 |
| Manufacture | 57.48 (40.65, 72.85) *** | 0.15 | 0.00 (-0.01, 0.01) | 0.01 | 0.13 (0.06, 0.19) *** | | 0.07 |
| GINI | 4148.22 (643.25, 7942.55) * | 0.05 | -0.97 (-2.68, 0.66) | -0.03 | -35.15 (-47.80, -22.19) *** | | -0.09 |
| Administration | -57.44 (-130.52, 23.40) | -0.03 | -0.07 (-0.11, -0.02) ** | -0.08 | -0.28 (-0.52, 0.03) * | | -0.03 |
| Median Income | -67.41 (-178.44, 25.73) | -0.03 | -0.06 (-0.11, -0.02) ** | -0.08 | -2.40 (-2.81, -2.02) *** | | -0.25 |
| Outlying | -396.75 (-580.28, -248.60) *** | -0.05 | 0.05 (-0.07, 0.17) | 0.02 | 1.77 (1.13, 2.60) *** | | 0.05 |
| Rural | -41.01 (-242.29, 169.94) | -0.01 | 0.11 (-0.01, 0.23). | 0.05 | 1.79 (0.95, 2.72) *** | | 0.06 |
| Without Insurance | -16.94 (-53.13, 20.51) | -0.03 | 0.03 (0.01, 0.04) *** | 0.12 | 0.19 (0.06, 0.32) ** | | 0.07 |
| Male | 191.68 (125.17, 251.45) *** | 0.16 | -0.04 (-0.06, -0.02) *** | -0.08 | 0.23 (0.03, 0.47) * | | 0.04 |
| Age over 65 | -74.08 (-98.63, -54.02) *** | -0.12 | 0.05 (0.04, 0.06) *** | 0.19 | 0.26 (0.15, 0.37) *** | | 0.09 |
| Population Density | 137.22 (-242.68, 1037.22) | 0.01 | 0.68 (0.49, 1.23) ** | 0.11 | -1.86 (-8.91, -0.79) | | -0.02 |
| Democrats | -52.42 (-63.32, -40.56) *** | -0.28 | -0.00 (-0.01, 0.01) | -0.00 | -0.32 (-0.38, -0.28) *** | | -0.36 |

Note: This table is analogous to Table 4 in the main text except the mediator is visit change (%).
