# Supplementary material for: Do racial and ethnic disparities in following stay-at-home orders influence COVID-19 health outcomes? A mediation analysis approach
Source: PLoS One. 2021 Nov 11;16(11):e0259803. doi: 10.1371/journal.pone.0259803 (PMC8584966; doi:10.1371/journal.pone.0259803)
Supplement: S4 Table — (DOCX) [file pone.0259803.s006.docx]

**Table S4. Output of Mediation Effects (Mediator: Visit change (%))**

|  |  |  | **Outcome** | | | | | |
| --- | --- | --- | --- | --- | --- | --- | --- | --- |
|  |  |  | Cases/100,000 | | | Deaths/100 cases | | |
| **Predictor** | |  | Est. (95% CI) | P-value | Std. Est. | Est. (95% CI) | P-value | Std. Est. |
| African American | | Total effect | 45.73*** (33.91, 56.73) | 0.00 | 0.24 | 13.92*** (7.81, 19.09) | 0.00 | 0.18 |
|  |  | Direct effect | 46.05*** (33.70, 57.55) | 0.00 | 0.24 | 13.79*** (7.72, 18.90) | 0.00 | 0.18 |
|  |  | Indirect effect | -0.32  (-1.17, 0.23) | 0.42 | -0.00 | 0.13  (-0.26, 0.66) | 0.52 | 0.00 |
| Hispanic | | Total effect | 74.08*** (64.26, 88.03) | 0.00 | 0.37 | 3.84  (-1.17, 9.62) | 0.15 | 0.05 |
|  |  | Direct effect | 73.45*** (62.90, 87.53) | 0.00 | 0.37 | 4.10  (-0.95, 10.00) | 0.12 | 0.05 |
|  |  | Indirect effect | 0.63  (-0.69, 2.06) | 0.34 | 0.00 | -0.25  (-1.07, 0.37) | 0.47 | -0.00 |
| Asian | | Total effect | 4.87  (-27.15, 45.05) | 0.79 | 0.00 | 24.15* (-1.40, 44.32) | 0.04 | 0.05 |
|  |  | Direct effect | 2.81  (-29.30, 41.95) | 0.87 | 0.00 | 24.98* (-0.16, 44.90) | 0.03 | 0.05 |
|  |  | Indirect effect | 2.06  (-1.81, 6.68) | 0.34 | 0.00 | -0.83  (-4.18, 1.06) | 0.48 | -0.00 |
| Other minorities | | Total effect | 56.82*** (37.37, 73.06) | 0.00 | 0.17 | 0.19  (-7.35, 4.76) | 0.95 | 0.00 |
|  |  | Direct effect | 56.55*** (36.75, 73.07) | 0.00 | 0.17 | 0.30  (-7.35, 4.75) | 0.93 | 0.00 |
|  |  | Indirect effect | 0.27  (-0.28, 1.31) | 0.48 | 0.00 | -0.11  (-0.61, 0.24) | 0.55 | -0.00 |

Note: This table is analogous to Table 5 in the main text except the mediator is visit change (%).
